# Therapy of childhood acute lymphoblastic leukemia in resource-poor geospaces

**DOI:** 10.3389/fonc.2023.1187268

**Published:** 2023-06-16

**Authors:** Moisés M. Gallardo-Pérez, Robert Peter Gale, Oscar A. Reyes-Cisneros, Daniela Sánchez-Bonilla, José A. Fernández-Gutiérrez, Wendy Stock, Iván Murrieta-Álvarez, Juan Carlos Olivares-Gazca, Guillermo J. Ruiz-Delgado, Rafael Fonseca, Guillermo J. Ruiz-Argüelles

**Affiliations:** ^1^ Centro de Hematología y Medicina Interna, Clínica Ruiz, Puebla, Puebla, Mexico; ^2^ Universidad Popular Autónoma del Estado de Puebla, Facultad de Medicina, Puebla, Puebla, Mexico; ^3^ Centre for Haematology, Imperial College of Science, Technology and Medicine, London, United Kingdom; ^4^ Universidad Anáhuac, Facultad de Medicina, Puebla, Puebla, Mexico; ^5^ Department of Medicine, University of Chicago, Chicago, IL, United States; ^6^ Laboratorios Ruiz, SYNLAB, Puebla, Puebla, Mexico; ^7^ Mayo Clinic, Division of Hematology-Oncology, Scottsdale, AZ, United States

**Keywords:** acute lymphoblastic leukaemia, resource poor countries, Total XI St. Jude protocol, outpatient, children

## Abstract

The therapy of children with acute lymphoblastic leukemia (ALL) in limited resource geospaces is challenging and must balance safety, efficacy, availability, and affordability. We modified the control arm of the St. Jude Total XI protocol for outpatient delivery including once-weekly daunorubicin and vincristine in initial therapy, postponing intrathecal chemotherapy until day 22, prophylactic oral antibiotics/antimycotics, use of generic drugs, and no central nervous system (CNS) radiation. Data were interrogated from 104 consecutive children ≤12 years (median, 6 years [interquartile range (IQR), 3, 9 years]. All therapies were given in an outpatient setting in 72 children. Median follow-up is 56 months (IQR 20, 126 months). A total of 88 children achieved a hematological complete remission. Median event-free survival (EFS) is 87 months [95% confidence interval (CI), 39, 60], 7.6 years in low-risk children (3.4, 8 years) whereas 2.5 years (1, 10 years) in high-risk children. The 5-year cumulative incidence of relapse (CIR) is 28% (18, 35%), 26% (14, 37%) in low-risk children and 35% (14, 52%) in high-risk children. Median survival for all subjects is not reached but must exceed 5 years. A total of 36 children relapsed at a median of 12 months (5, 23 months). Outcomes were comparable to those reported in the control arm of the Total Therapy XI study, but inferior to current treatment protocols in high-income countries. The average cost of the first 2 years of therapy was $28,500 USD compared with an average cost of approximately $150,000 USD in the US, an 80% saving. In conclusion, using an outpatient-based modification of the St. Jude Total XI protocol, we obtained good results with relatively few hospitalizations or adverse events and at a substantial saving. This model can be applied in other resource-poor geospaces.

## Introduction

Long-term survival is achieved in more than 90% of children <10 years old with acute lymphoblastic leukemia (ALL) (1). In resource-poor geospaces, the selection of chemotherapy regimens is challenging and must balance safety, efficacy, availability, and affordability. We modified the control arm of the St. Jude Total XI protocol for outpatient delivery (2–7) and reported outcomes in 104 consecutive children with ALL. We compared our results with those obtained in other geospaces and employing other treatment schedules.

## Methods

### Subjects

All consecutive children 0–12 years with ALL diagnosed and treated in the Centro de Hematología y Medicina Interna (HMI) de Puebla from September 1983 to 2022 and completed therapy were included. A total of 10 subjects were excluded for not completing therapy because they were lost to follow-up. HMI is a private practice center located in Puebla, México, where people pay for treatment out of pocket or are supported by philanthropies.

### Diagnosis

Blood and bone marrow smears were stained with May–Grümwald–Giemsa and classified according to the FAB classification (1). The immune phenotype and DNA content were analyzed by flow cytometry (8, 9). Cytogenetics were done by conventional techniques. *BCR::ABL1* transcripts (after March 1994) and *IKAROS* mutations (after July 2014) were assessed by multiplex reverse transcribed polymerase chain reaction (RT-PCR) (10–12).

### Therapy

Subjects received a modified control arm of the St. Jude Total XI regimen, which was chosen because it uses inexpensive generic drugs available in México and, with modifications, can be given in an outpatient setting (5–7): Modifications included initial weekly dosing, postponing intrathecal chemotherapy until day 22 to avoid contamination of the cerebrospinal fluid with blast cells from the peripheral blood, prophylactic oral antibiotics/antimycotics, no CNS radiation, and placement of a central IV catheter. **Table 1** shows the main characteristics of modified St. Jude TOTAL XI regimen.

**Table 1:** Drugs, doses, and schedule used in the modified St. Jude TOTAL XI regimen.

Induction		
	Route	Dose
Prednisone	Oral	40 mg/mE+2/d × 28 days
Vincristine	IV	1.5 mg/mE+2 days 1, 8, 15, and 22
Daunorubicin	IV	25 mg/mE+2, days 1, 8, and 15
L-asparaginase	IM	10,000 U/mE+2 days 4, 10, 15, and 19,
Etoposide	IV	200 mg/mE+2 days 22 and 29
Cytarabine	IV	300 mg/mE+2 days 22, 25, and 29
Consolidation		
Methotrexate	IV	2 g/mE+2 by 2 h IV infusion days 44 and 51
Folinic acid	Oral	10 mg/mE+2 every 4 h × 12 doses
Maintenance therapy ^a)^		
6-Mercaptopurine	Oral	50 mg/mE+2/day
Methotrexate	Oral	20 mg/mE+2/week
Intrathecal therapy		
**Drug**	**Dose**	
Methotrexate	12 mg/mE+2	Days 22 and 43 during induction therapy and every 8 weeks during maintenance therapy for 2 years
Dexamethasone	4 mg/mE+2	
Cytarabine	25 mg/mE+2	

a Oral methotrexate rounded to the next full dose according to tablet size with monthly pulses of rotating combinations of the same drugs used for induction (vincristine, daunomycin, and asparaginase × 3 weekly doses, etoposide and cytarabine × 2 weekly doses; and MTX + folic acid × 1).

**Figure 1:**
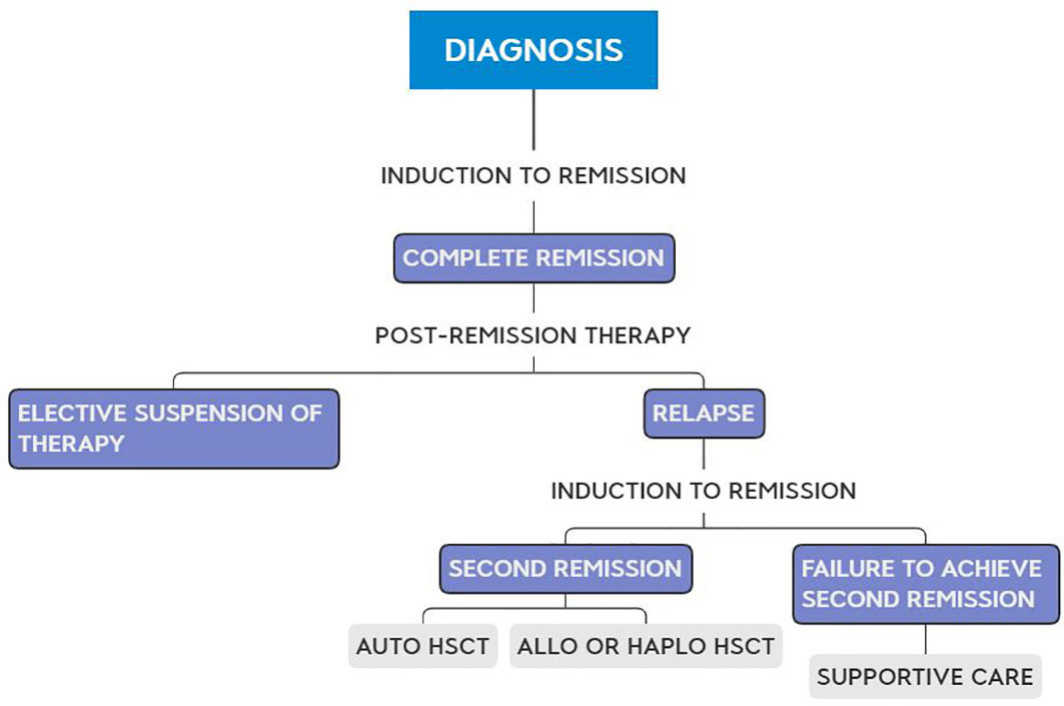
Flow chart of the employed treatment.

Therapy was given in an outpatient setting. After June 2001, children with *BCR::ABL1* received imatinib, 400 mg/day, or dasatinib, 100 mg/day, 7 days after starting therapy and were assessed for hematopoietic cell transplant after achieving a complete remission. The average cost of the first 2 years of therapy was calculated, employing the costs of the drugs and the medical fees for a child with a body surface area of 1 mE+2, treated fully on an outpatient basis. **Figure 1** depicts the flow chart of the employed treatment.

### Definitions

Hematological complete remission was defined as no lymphoblasts in blood and cerebrospinal fluid (CSF) and ≤5% in bone marrow for ≥30 days, normal bone marrow histology, no signs or symptoms of leukemia, ECOG performance score of 0–1, and ≥0.5 × 10E+9/L granulocytes and ≥50 × 10E+9/L platelets. Low risk was defined as all of the following: age 1–10 years, white blood cells (WBCs) at diagnosis <50,000 and B phenotype. High risk was defined as one or more of the following: 1) age < 1 or > 10 years; 2) WBC > 50 × 10E+9/L; 3) non-B-cell phenotype; 4) Ph^1^-chromosome- or *BCR::ABL1*-positive; 5) *IKAROS* mutation; and/or 6) t (4, 11). CNS leukemia was diagnosed based on cranial nerve palsies with or without leukemia blasts in the CSF or when mononuclear cells were ≥ 5 × 10E+6/L with leukemia blasts seen on cyto-centrifuged slides. The diagnosis of testes leukemia required biopsy. After 1998, measurable residual disease (MRD) was assayed at the end of induction and consolidation therapies by multi-parameter flow cytometry (MPFC) (12) or PCR (9) and repeated every 3 months whilst receiving therapy. Induction therapy was restarted when the MRD test became positive (>1 × 10E−5 nucleated cells) and a transplant planned after achieving a second complete remission.

### Statistics

The primary endpoint of the analysis was event-free survival (EFS) defined as the interval from starting therapy to relapse or death from any cause. Cumulative incidence of relapse (CIR) was defined as the probability of relapse in children achieving a hematological complete remission. Continuous complete remission (CCR) was defined as the interval from complete remission to relapse. Survival was defined as the interval from starting therapy to death. Transplant recipients were censored at transplant. EFS, CIR, and survival were estimated by Kaplan–Meier plots. Outcomes in the low- and high-risk cohorts were compared using a two-sided log-rank test. Statistical analysis was performed using R Statistical Software (version 3.6.1; R Foundation for Statistical Computing, Vienna, Austria).

## Results

A total of 104 consecutive children were analyzed. Median age is 6 years (3–9 years). Of the children, 100 had B-cell lineage, 3 had T-cell lineage, and 1 had null-ALL. At diagnosis, 82 had a WBC concentration <20 × 10E+9/L, 9 had 20–50 × 10E+9/L, and 13 had >50 × 10E+9/L. The DNA content, analyzed in 38 children, was diploid in 22, hyper-diploid in 14, and hypo-diploid in 2. Five of the 56 children tested were *BCR : ABL1* positive, and two of the six were *IKAROS* mutated.

**Figure 2:**
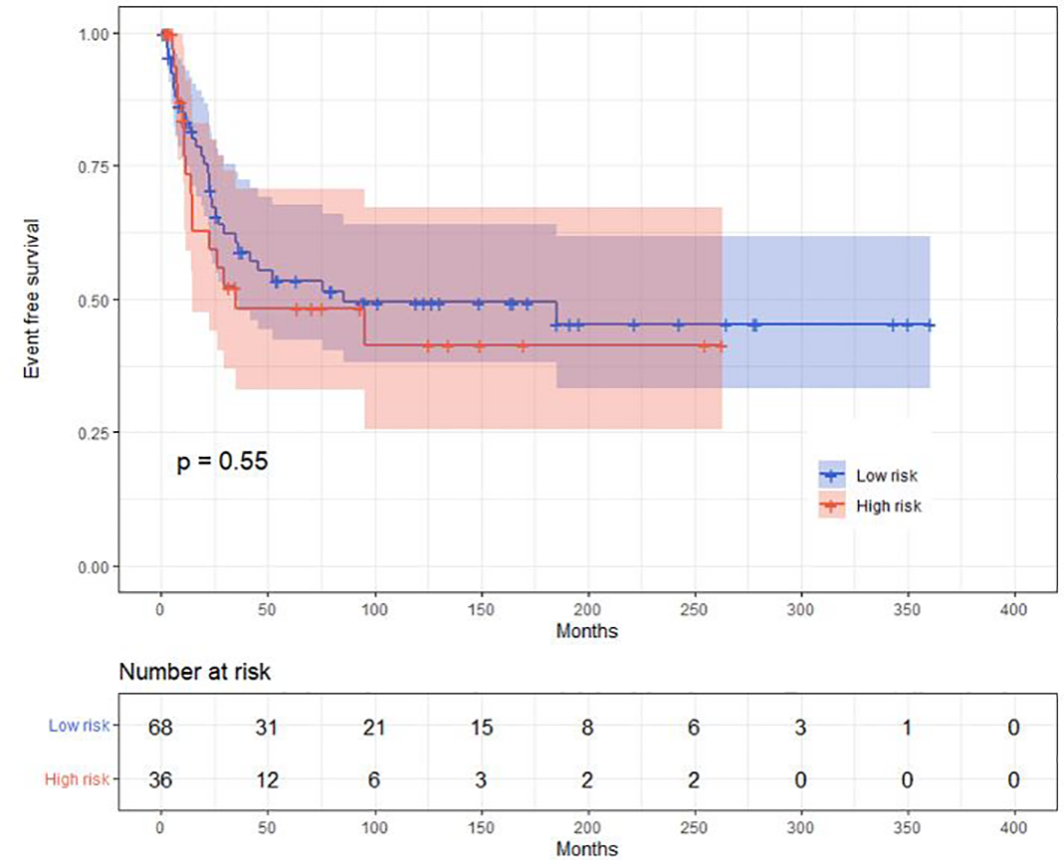
Event-free survival curve in the low- and high-risk cohorts.

**Figure 3:**
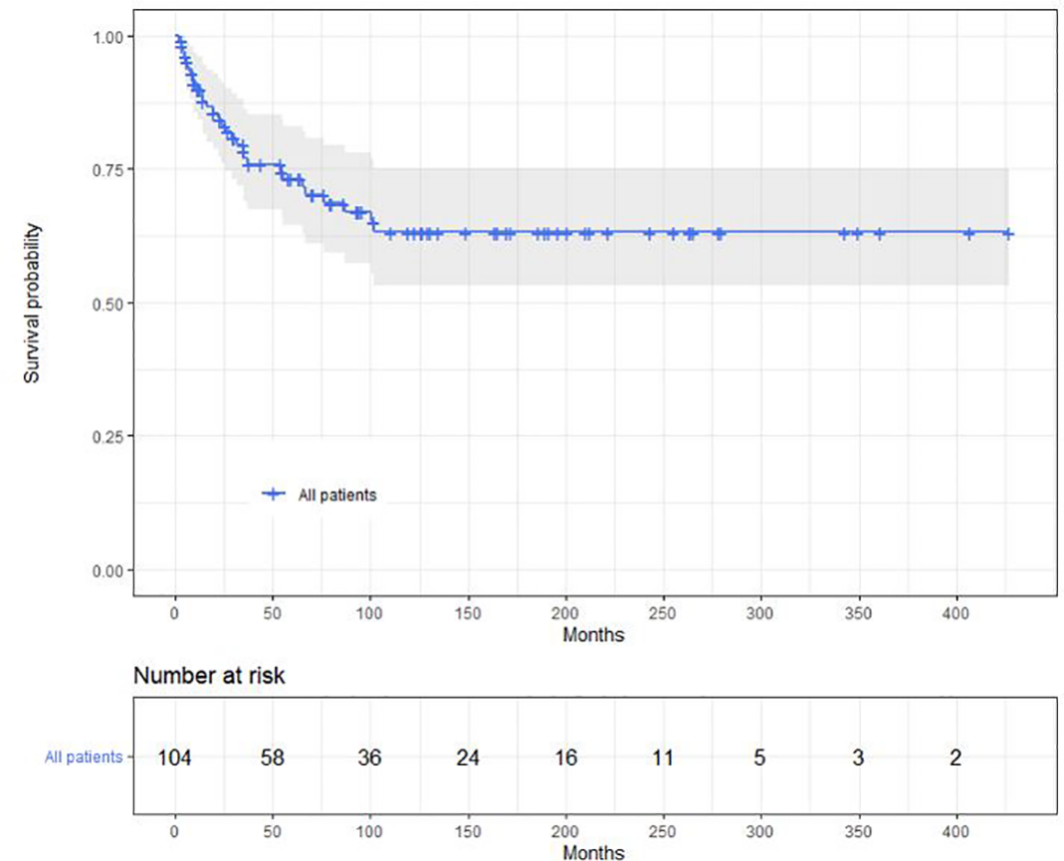
Overall survival of the 104 children.

**Figure 4:**
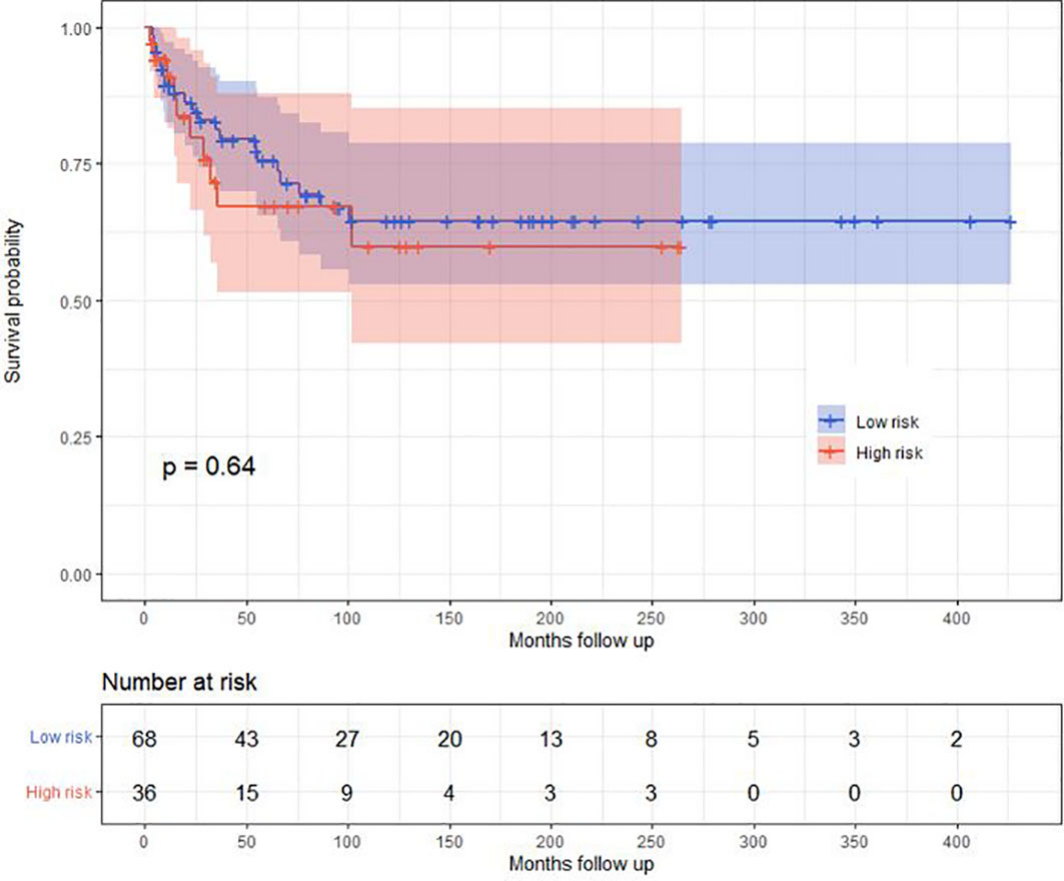
Overall survival of the low- and high-risk cohorts.

Median follow-up is 56 months (IQR, 20, 12 months). A total of 88 children achieved a hematological complete remission including 57 in the low- and 31 in the high-risk cohorts (*p* = 0.001). A total of 36 children subsequently relapsed, 18 in the bone marrow only, 13 in the CNS only, 5 in both, and 4 with a synchronous testes relapse. 74 children received chemotherapy only, and 30 children received an autologous (N = 5), allogeneic (N = 23), or auto-allo (N = 2) transplant. Autologous transplants were done in children lacking an HLA-identical sibling. There were 36 children in the low- and 68 in the high-risk cohort. Median time to relapse was 17 months (95% CI, 5, 33 months). Median EFS is 87 months (39, 60 months). Median EFS in the low-risk cohort is 92 versus 35 months (13, 129 months) in the high-risk cohort (*p* = 0.55; **Figure 2**). CIR at 5 years is 28% (18, 35%), 26% (14, 37%) in the low- and 35% (14, 52%) in the high-risk cohorts (*p* = 0.74). Median survival has not been achieved, being >5 years (**Figure 3**). In the low-risk cohort, 20-year survival probability is 66% (55, 80%) versus 61% (45, 86%; *p* = 0.64) in the high-risk cohort (**Figure 4**).

Chemotherapy was started in an outpatient setting in all subjects. Four were hospitalized in the first 7 weeks for mucositis, granulocytopenia, meningitis (two patients), and 27 were hospitalized thereafter at a median of 10 days (8–13) after starting therapy. The leading causes of hospitalization were meningitis, mucositis, and pneumonia. A total of 72 children had all their therapy as outpatients.

## Discussion

Data from our study indicate success in treating children with ALL in a resource-poor geospace by modifying the St. Jude Total Therapy XI study for a predominantly outpatient setting, using inexpensive generic drugs and adjusted doses. Of the children, 70% were never hospitalized. Outcomes were like those reported for the control arm of the Total Therapy XI study (6, 7, 14) but inferior to current treatment protocols in high-income countries. The average cost of the first 2 years of therapy of a child with a body surface area of 1 mE+2 and treated fully as outpatient and without complications was $28,500 USD compared with an average cost of $150,000 USD in the US, an 80% saving (14). This cost does not include non-medical cost not incurred at the clinic, such as transportation and accommodation out of the clinic and tutor or parent time off from employment. It is also important to consider that the cost of the drugs varies and that if the costs were more effectively controlled, more children might receive treatment. The need of data management programs for careful documentation of medical costs should also be considered.

In adolescents and young adults, we have previously shown that our TOTAL-XI-based chemotherapy schedule is more effective and less toxic than the Hyper-CVAD regimen, which is commonly employed in North America and requires inpatient chemotherapy administration (4, 5). The 5-year OS in children with ALL treated in different regions of the world ranges from 8% in Eastern Africa to 83% in North America, Latin America being approximately 50% (14–23) (see **Supplementary Table S1**). Thus, our results over a 30-year period suggest slightly better outcomes than those previously reported in Latin America studies and similar to those obtained in other middle income countries.

Our study has several limitations. First, it was done over 39 years, over which time technologies such as *BCR::ABL1*-testing, diagnostics, supportive care, financial conditions, and therapies such as TKIs have evolved. Second, we lacked cytogenetic and mutation analyses for some children. Third, substantial numbers of children were lost to follow-up, a common problem in resource-poor geospaces (17, 18). Fourth, the value of currency has changed in our country during the study.

In conclusion, using an outpatient-based modification of the St. Jude Total XI protocol, we obtained good results with relatively few hospitalizations or adverse events and at a substantial saving. This model can be applied in other resource-poor geospaces, with difficulties to admit patients to the hospital.

## Data availability statement

The raw data supporting the conclusions of this article will be made available by the authors, without undue reservation.

## Ethics statement

The studies involving human/animal participants were reviewed and approved by Comité de Ética en Investigación del Centro de Hematología y Medicina Interna, Clínica Ruiz. The patients/participants provided their written informed consent to participate in this study.

## Author contributions

Conception and design: GR-A and GR-D. Collection and assembly of data: OR-C, DS-B, JF-G, IM-Á, and JO. Data analysis and interpretation: WS, RF, RG, MG-P, and GR-A. Manuscript writings: All authors. The work reported in the paper has been performed by the authors, unless clearly specified in the text. All authors contributed to the article and approved the submitted version.
