# Supplementary material for: Therapy of childhood acute lymphoblastic leukemia in resource-poor geospaces
Source: Front Oncol. 2023 Jun 16;13:1187268. doi: 10.3389/fonc.2023.1187268 (PMC10312136; doi:10.3389/fonc.2023.1187268)
Supplement: Supplementary file 1 [file Table_1.docx]

**Table S1. Differences in treatment results between low-, middle- and**

**high-income countries.**

| Region | Results |
| --- | --- |
| This study  (Middle income country) | |
| Complete Remission Rate | **84%** |
| Relapse rate | **34%** |
| Overall Survival (>5 years) | **77%** |
| Africa  (Low-income countries) [19,20] | |
| Complete Remission Rate | 76% |
| Relapse rate | 27% |
| Overall Survival (>5 years) | 63% |
| China  (Middle-income countries) [21] | |
| Complete Remission Rate | 98% |
| Relapse rate | 7.1% |
| Overall Survival | 90% |
| USA and Europe  (High-income countries) [22,23] | |
| Complete Remission Rate | >90% |
| Relapse rate | 20% |
| Overall Survival (>5 years) | 80% |
